# The impact of mental state altering medications on preventable falls after total hip or total knee arthroplasty: a systematic review and meta-analysis

**DOI:** 10.1186/s13037-023-00387-4

**Published:** 2024-02-12

**Authors:** Elsbeth J. Wesselink, Marinus van der Vegt, Sharon Remmelzwaal, Sebastiaan M. Bossers, Eric J. Franssen, Eleonora L. Swart, Christa Boer, Marcel A. de Leeuw

**Affiliations:** 1 Department of Clinical Pharmacy, Zaans Medisch Centrum, Koningin Julianaplein 58, 1502 DV Zaandam, The Netherlands; 2 Department of Anaesthesiology, Zaans Medisch Centrum, Zaandam, The Netherlands; 3 Epidemiology & Data Science, Amsterdam UMC location Vrije Universiteit Amsterdam, 1117 Boelelaan, Amsterdam, The Netherlands; 4 Amsterdam UMC location Vrije Universiteit Amsterdam, 1117 Anesthesiology, Boelelaan, Amsterdam, The Netherlands; 5 Department of Clinical Pharmacy, OLVG Hospital, Amsterdam, The Netherlands; 6 Pharmacy and Clinical Pharmacology, Amsterdam UMC location Vrije Universiteit Amsterdam, 1117 Boelelaan, Amsterdam, The Netherlands; 7 Faculty of Medicine, Amsterdam UMC location Vrije Universiteit Amsterdam, 1117 Boelelaan, Amsterdam, The Netherlands

## Abstract

**Background:**

Joint replacement surgery of the lower extremities are common procedures in elderly persons who are at increased risk of postoperative falls. The use of mental state altering medications, such as opioids, antidepressants or benzodiazepines, can further contribute to impaired balance and risk of falls. The objective of the current systematic review was to evaluate the risk of the use of mental state altering medications on postoperative falls in patients undergoing total hip arthroplasty (THA) or total knee arthroplasty (TKA).

**Methods:**

A comprehensive search of Medline, Embase and Cochrane Controlled Trials Register was conducted from 1 October 1975 to 1 September 2021. The search was repeated in may 2023 and conducted from 1 October 1975 to 1 June 2023. Clinical trials that evaluated the risk of medication on postoperative THA and TKA falls were eligible for inclusion. Articles were evaluated independently by two researchers for risk of bias using the Newcastle-Ottawa Scale. A meta-analysis was performed to determine the potential effect of postoperative use of mental state altering medications on the risk of falls. Lastly, a qualitative synthesis was conducted for preoperative mental state altering medications use.

**Results:**

Seven cohort studies were included, of which five studies focussed on the postoperative use of mental state altering medications and two investigated the preoperative use. Meta-analysis was performed for the postoperative mental state altering medications use. The postoperative use of mental state altering medications was associated with fall incidents (OR: 1.81; 95% CI: 1.04; 3.17) (p < 0.01) after THA and TKA. The preoperative use of opioids > 6 months was associated with a higher risk of fall incidents, whereas a preoperative opioid prescription up to 3 months before a major arthroplasty had a similar risk as opioid-naïve patients.

**Conclusions:**

The postoperative use of mental state altering medications increases the risk of postoperative falls after THA and TKA. Prior to surgery, orthopaedic surgeons and anaesthesiologists should be aware of the associated risks in order to prevent postoperative falls and associated injuries.

## Background

Pain induced by arthrosis often results in total hip arthroplasty (THA) and/or total knee arthroplasty (TKA) since this is a successful surgery with strong evidence for improving physical function and health-related quality of life. Fast track procedures in an ageing population are widely implemented over the past 20 years, and have proven effective in terms of length of stay (LOS), complications and readmission rates [1, 2]. Outpatient TKA/THP with a maximum of 24 h observation if needed is largely performed in the United States, and as a result the main part of the rehabilitation period takes place outside the hospital. However, within the first few weeks after surgery, patients are at increased risk of falling since early and persistent muscle loss results in impaired balance and poor walking ability [3–5]. Periprosthetic Fractures have a major impact in community health care resources since these are associated with higher rates of morbidity and mortality [6].

In addition to joint problems, age and multiple comorbidities may influence the risk of complications like falling after TKA and THA [7, 8]. People aged 65 years and older have the highest risk of falling, with 30% of people older than 65 years and 50% of people older than 80 years falling at least once a year. Falls are the main cause of accidental death in persons aged 65 years and older, the cumulative mortality after 1 year of a hip fracture ranges between 20 and 40% [4, 9–11]. The number of comorbidities increases with age, Parkinson’s disease, hypotension, rheumatic disease, history of stroke, depression, cognitive impairment, diabetes and pain have strong associations with falling [4, 5, 12–14]. Furthermore, the pharmacological treatment of these comorbidities, including antidepressants, antiepileptics, antihypertensives, benzodiazepines and opioids, often affects the Central Nervous System (CNS) and mental state. The side effects of most mental state altering medications include drowsiness, dizziness and cognitive and motor impairment. The use of mental state altering medications may also increase the risk of falling [5, 7]. The aim of this review was to perform a systematic review of available literature from clinical trials with regard to fall incidents related to mental state altering medications after THA and TKA.

## Methods

### Protocol and registration

This systematic review and meta-analysis was conducted following the guidelines of the Preferred Reporting Items for Systematic Reviews and Meta-analyses (PRISMA) [15]. The protocol of the current review was registered on the International Prospective Register of Systematic Reviews (PROSPERO; CRD42022316802).

### Search strategy

A comprehensive search using the electronic databases of MEDLINE, EMBASE, and the Cochrane Controlled Trials Register was conducted from 1 October 1975 to 1 September 2021. The search was repeated in may 2023 and conducted from 1 October 1975 to 1 June 2023. The search consisted of the following terms used in the title or abstract, or as MeSH terms: “falls”, “hip replacement”, “knee replacement”, “arthroplasties”, “knee replacement”, “arthroplasty”, “hip replacement”, “accidental”. The reference lists of the studies examined to determine study eligibility were hand-searched for relevant studies.

Eligibility criteria included prospective and retrospective study designs, with adult participants of both sexes who underwent THA or TKA. Postoperative falls and the systemic use of medication of the Central Nervous System were reported in the study. Studies that investigated local administration of analgesics or anaesthetics were excluded.

### Study identification

The principal author (EW) conducted the database search. The principal author and one independent reviewer (SB) screened titles and abstracts to determine eligibility for inclusion. The decision to include or exclude studies, based on the eligibility criteria, was made independently by each author. Full-texts of potentially relevant studies were retrieved for further assessment. Discrepancies in opinions were resolved by a third senior reviewer (RV).

### Risk of bias assessment

The quality of studies was independently evaluated by two reviewers (EW and SR). The Newcastle-Ottawa Scale (NOS) tool was used to evaluate the non-randomized studies [16]. The NOS is a valid and reliable tool that contains in total eight methodological items in three domains with a maximum score of nine points. A higher score indicates a lower risk of bias, studies with a NOS score ≥ 5 were included in the systematic review.

### Data extraction

The principal author (EW) extracted relevant data from individual studies and the data were independently checked by a second reviewer (SR) for consistency. Extracted data included study characteristics such as study design, participant numbers, treated condition, follow-up period, number of falls and findings. When the values of standard deviations (SD) were not provided for the intervention or control group, these were calculated from confidence intervals, and p-values for differences in means.

### Primary outcome

Postoperative fall incidents after THA or TKA was the primary outcome for this systematic review. The method of evaluation for each study is stated in the relevant tables.

### Statistical analysis

Studies were meta-analysed by using a random-effects model when more than three studies reported number of fall incidents for both the experimental (mental state altering medications use) and control (no mental state altering medications use) groups. A pooled odds ratio was computed and heterogeneity was assessed by I^2^ and τ^2^. All analyses and plots were performed in RStudio 4.0.3 using the ‘meta’ package [17]. Studies with the right outcome, study design and intervention but preoperative use of mental state altering medications were reported qualitatively.

## Results

**Fig. 1:**
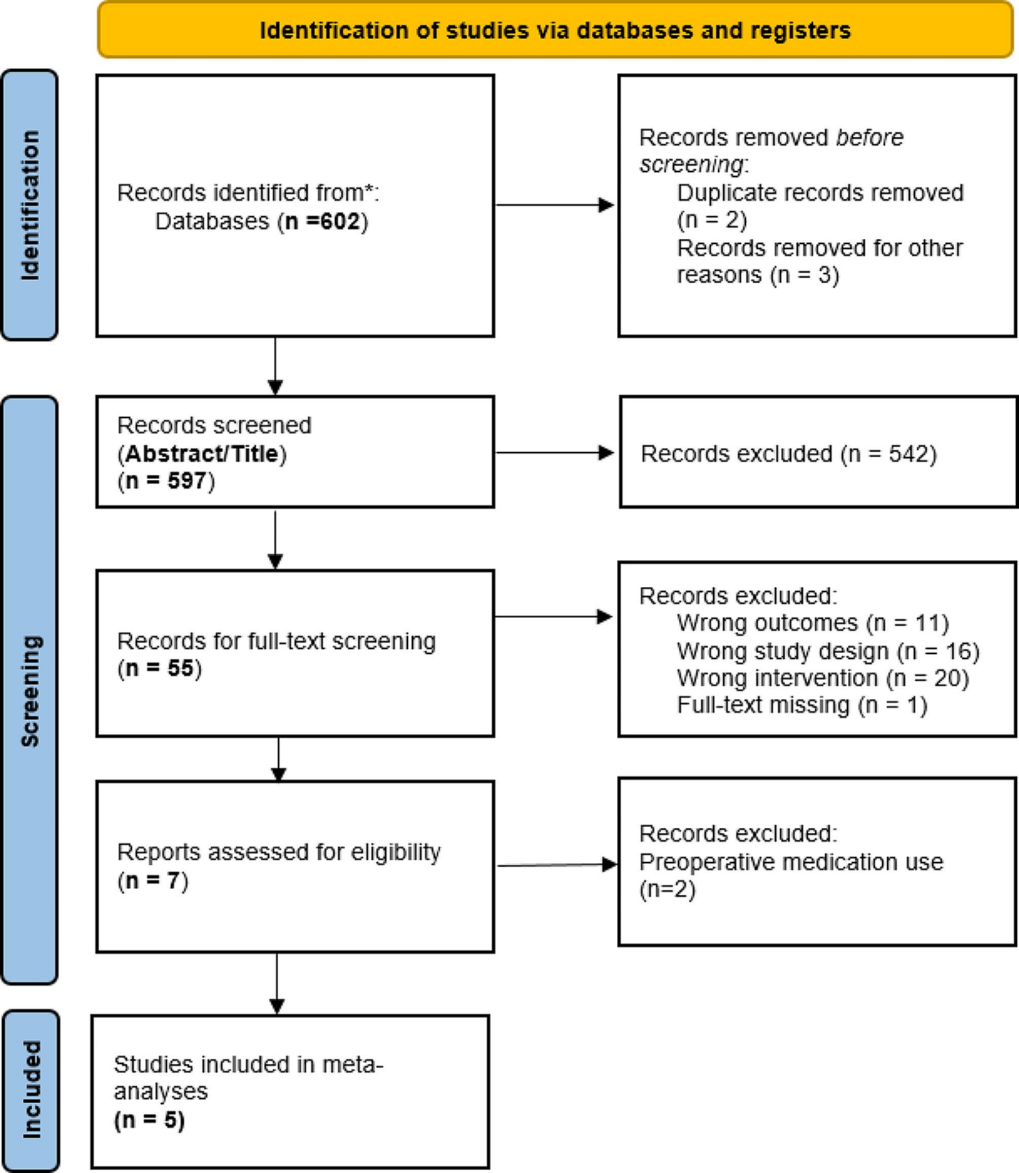
PRISMA flow chart of study selection process

The database searches resulted in 602 titles (Fig. 1). Following the removal of duplicates and exclusions of records based on title and abstract screening, a total of 60 studies were available for full-text review. A total of 53 studies were further excluded with inappropriate study design (predominantly review studies) or wrong outcomes, leaving a final selection of 7 studies meeting the inclusion criteria. Of the included studies, 5 studies had an adequate postoperative follow-up period for the use of mental state altering medications and were included in the meta-analysis (Table 1). The two other studies were reported qualitatively [18–24].

**Table 1:** Characteristics of studies and intervention details for rotator cuff calcifying tendinitis

Author year	Design	Setting	TKA/THA	Intervention	Most important Inclusion criteria	Sample size (n)	Intervention vs. Control (n)	Follow-up	Primary endpoint	Secondary endpoints	Patients with fall incident (%)	Mental state altering medications related fall incident (%)	Statistical analysis	NOS
Ardeljan, 2021 [23]	Retrospective matched-controlled	Insurance database	TKA	Postop Zolpidem	TKA	594.973 (374.681 female)	99.178 (cases) 495.795 (control)	90 days	Complications including falls	None	35.521 (5.9%)	Zolpidem user: 6486 (18,3%) No-zolpidem user: 29,035 (81,7%)	Multivariate logistic regression	7
Hill, 2021 [24]	Prospective observational cohort study	Single center	TKA	Postop CNS medications	TKA, > 60	251 (140 female)	N/A	12 months	Fall rate	Injurious falls, multiple falls	102 (40.6%)	Mental state altering medication-user: 47 (46.1%) No-mental state altering medication user: 55 (53.9%)	Logistic and negative binomial regression	6
Hill, 2021 [22]	Prospective observational cohort study	Single center	THA	Postop mental state altering medications	THA, > 60	167 (91 female)	N/A	12 months	Fall rate	Injurious falls, multiple falls	67 (40.1%)	Mental state altering medication user: 59 (88.1%) No-mental state altering medication user: 8 (11.9%)	Logistic and negative binomial regression	6
Jörgense, 2013 [21]	Prospective, descriptive, multicenter study	Danish National Patient registry	TKA/THA	Postop pharmacologically treated psychiatric disease	THA/TKA	5145 (2932 female)	N/A	90 days	Fall-related hospital admissions after fast-track	Risk factors for falls	92 (1.8%)	Antipsychotic user: 16 (17.4%) No-antipsychotic user: 76 (82.6%)	Univariate / multivariate logistic regression	5
Levinger, 2017 [20]	Prospective, observational	Single center	TKA/THA	Postop antidepressant	THA/TKA	243 (128 female)	N/A	12 months	Falls	Circumstances of falls	82 (33.7%)	Antidepressant user: 16 (19.5%) No-antidepressant user: 66 (80.5%)	Binary logistic regression	6

### Description of included studies

The mental state altering medications reported in the five studies in the meta-analysis included pharmacologically treated psychiatric disease. These were antidepressants, antipsychotics, benzodiazepines, opioids and neurological medication. Ardeljan et al. looked at the most described hypnotic for insomnia in the United States, zolpidem. Zolpidem use following TKA was in this study associated with a higher risk of morbidity and falls [23]. Participants in the studies of Hill et al. were categorized as being prescribed / not prescribed mental state altering medications including antidepressants, benzodiazepines, opioids or any neurological medication such as carbamazepine [22, 24]. Jorgensen included patients with pharmacologically treated psychiatric disease, whereas Leveringer solely looked at the of antidepressants after THA/TKA and the risk of falling [20, 21]. No studies reported about the dose, frequency or interactions of the mental state altering medications, but Hill analysed the effect of the number of mental state altering medications in use. For TKA this was reported as a risk factor for falling.

### Meta-analysis

**Fig. 2:**
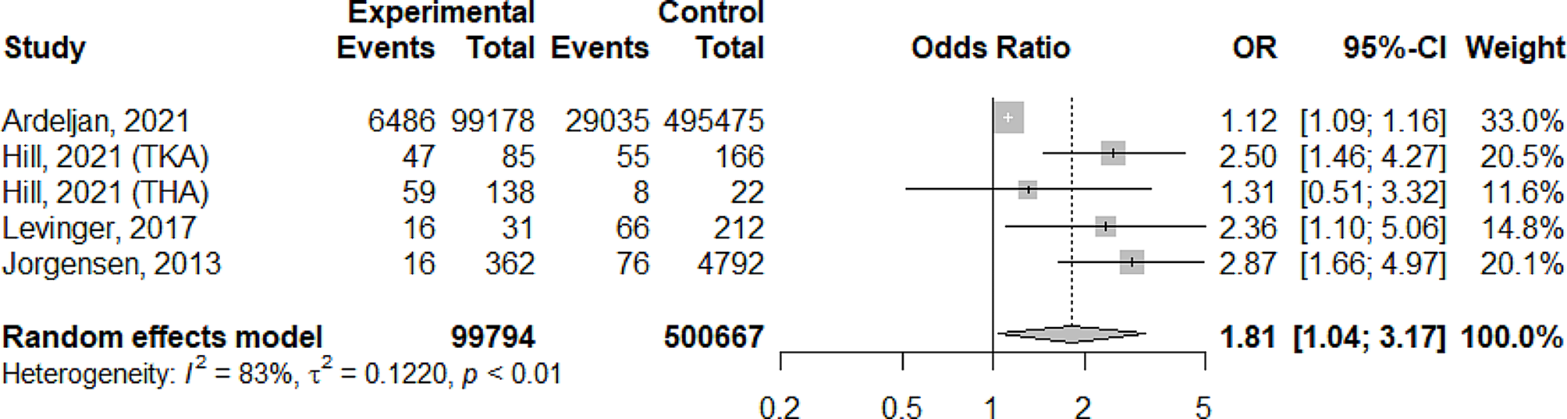
Forest plot of the effect of the use of mental state altering medications on fall incidents after THA and TKA

For all studies, the number of patients with a fall incident (fallers) and the use mental state altering medications were identified as the intervention group. The number of patients with a fall incident without the use of mental state altering medications were identified as the control group. Because some studies compared fallers with non-fallers, and identified risk factors for falling, the control groups in the meta-analysis may be relatively small. In all studies, increased odds were found for fall incidents in the group that postoperatively used mental state altering-medications compared to the group who did not use mental state altering-medications [20–24]. Overall, the meta-analysis shows an OR of 1.81 (95% CI 1.04–3.17) for the use of mental state altering medications after THA/TKA (Fig. 2).

### Qualitative analysis

Two studies reported about the association between preoperative opioid use and the risk of postoperative fall [18, 19]. Jain et al. concluded that the risk of falling within 90 days after TKA is significantly higher for the patients who had used opioids for > 6 months preoperatively as compared with the opioid-naïve patients. The association between opioid use and the risk of falling was not significant in the THA group. A 3 months prescription-free period before surgery mitigated this risk for chronic opioid users. However, Riddle et al. concluded that patients taking opioids were not at an elevated risk for falls following THA of TKA. Since postoperative use of opioids was not assessed in either studies, they were excluded from the meta-analysis.

### Quality assessment

The risk of bias assessment is summarized in Table 2. No differences in assessment between the two reviewers were observed. Selection and outcome criteria had a maximal score for all studies included, only Ardeljan et al. had a higher score on comparability and randomly matched study group patients to controls by age, gender, and comorbidities. Overall, the mean score ± SD of the NOS bias tool among the five studies was 6 ± 0.6, no studies with a NOS < 5 were assessed.

**Table 2:** Summary of critical appraisal of included studies using the newcastle-ottawa quality assessment scale for cohort studies

Study ID	Selection (max 4 stars)	Comparability (max 2 stars)	Outcome (max 3 stars)
Ardeljan (2021)	****	*	**
Hill, TKA (2021)	****	--	**
Hill, THA (2012)	****	--	**
Jörgensen (2013)	***	--	**
Levinger (2017)	****	--	**

*Selection*

1) Representativeness of intervention cohort– (a) truly representative of average TKA/THA patient *; (b) somewhat representative of average TKA/THA patient*; (c) only selected group of patients; no description of derivation of cohort

2) Selection of non-intervention cohort—a) drawn from same community as intervention cohort*; b) drawn from different source; c) no description of the derivation of the non-intervention cohort

3) Ascertainment of intervention—a) health record*; b) structured interview*, c) written self-report; d) no description

4) Demonstration that outcome was not present of start of study—a) yes*; b) no

*Comparability*

1) Comparability of cohorts on basis of design or analysis—a) study controls for age, sex or comorbidities*, b) study controls for any additional factors*

*Outcome*

1) Assessment of outcome—a) independent blind assessment*; b) record linkage*; c) self-report; d) no description

2) Was follow up long enough for outcomes to occur—a) yes*; b) no

3) Adequacy of follow up cohort—a) complete follow up*; b) minimal loss to follow up (≤20%); c) follow up rate < 80% and no description of losses to follow up; d) no statement

## Discussion

The aim of the current systematic review and meta-analysis was to evaluate the evidence for fall incidents related to the use of mental state altering medications after THA and TKA. We found a significant increase in fall incidents if mental state altering medications is used. This is in line with other studies reporting about increased fall incidents after TKA/THA or medication [14].

### Implications for practice

The results of the current systematic review and meta-analysis have important implications for practice. First, multifactorial risk assessment including the use of mental state altering medications of older frail people who are scheduled for TKA or THA is important to prevent postoperative fall incidents and associated injuries. Prior to surgery, orthopaedic surgeons and anaesthesiologists should be aware of the associated risks. Second, if mental state altering medications are used, the possibility of preoperative stop of this medication should be explored. The patient’s preferences should also be taken into account [25]. Third, since the rise of improved perioperative recovery protocols has led to shorter hospital stays for many patients, frail older patients with mental state altering medications are at increased risk of falling in case of early discharge. A prescription for new postoperative pain, including opioids, on top of the mental state altering medications already used for comorbidities, postoperative muscle weakness and disbalance are risk factors for falling. These factors should be considered by a multidisciplinary team including a geriatrician and a pharmacist and may be a contraindication for fast track surgery.

### Future recommendations

The results of the current systematic review have several implications for future research. First, preoperative opioid use over > 6 months is associated with increased fall incidents after TKA, but data on the effect of postoperative use of opioids versus opioid-free analgesia on fall incidents are generally lacking [18, 19]. Second, in an outpatient setting deterioration of side effects may remain unnoticed and may increase the risk of fall incidents. We encourage future prospective studies to evaluate patient-controlled pain management on the risk of falling in an outpatient setting.

Third, patients with polypharmacy may benefit from multidisciplinary attention in an inpatient setting to prevent postoperative risk on falling. Several studies have previously investigated the effectiveness of a structured medication review with varying success depending on the selected setting, subgroup of patients and outcome measure [26–29]. In an inpatient setting medication reviews are not common practice yet. Multidisciplinary triage around TKA/THA surgery may detect unbalanced frail older patients in their response on combined medication. Therefore, we encourage prospective studies to evaluate the effect of inpatient multidisciplinary medication reviews on clinical outcomes after TKA/THA. Preferably, the medication reviews are performed soon after the orthopaedic surgeon’s decision for TKA/THA.

### Limitations of the study

Our aim was to include studies on all kinds of mental state altering medications that had been used before or after TKA and THA and reported fall incidents. Therefore, we conducted a comprehensive search strategy by including all possible synonyms to avoid missing any potential relevant trials, hence reducing publication bias. To date, we are not aware of any previous systematic reviews that evaluated the use of mental state altering medications and the association with fall incidents after TKA and THA.

The heterogeneity of 83% between the studies can be explained by several differences in the design of the studies. First of all, studies with THA and TKA are included. Although the risk of falling is increased after both types of total joint surgery, the pain level and revalidation period is different. Second, the follow up period varies from 3 to 12 months between the studies. The highest risk of falling related to revalidation and surgery is within the first months after surgery. Thereafter, new or other causes of falling could have played a role. Although well defined in the methods and considered in the NOS score, the studies of Hill and Jörgenson are limited by the fact that the assessment of fall incidents was done by the less reliable method of self-reporting.

## Conclusions

The postoperative use of mental state altering medications increases the risk of postoperative falls after THA and TKA. High heterogeneity between the studies limited the conclusions of the present systematic review.

## Data Availability

The datasets used and/or analysed during the current study are available from the corresponding author on reasonable request.

## Declarations

### Authors' information

This study is part of the PhD project of EW entitled “Perioperative medication management”

## Abbreviations

CNS: Central Nervous System
LOS: Length of stay
NOS: Newcastle-Ottawa Scale
THA: Total Hip Arthroplasty
TKA: Total Knee Arthroplasty
